# Evidence of functional cell-mediated immune responses to nontypeable *Haemophilus influenzae* in otitis-prone children

**DOI:** 10.1371/journal.pone.0193962

**Published:** 2018-04-05

**Authors:** Elke Seppanen, Dino Tan, Karli J. Corscadden, Andrew J. Currie, Peter C. Richmond, Ruth B. Thornton, Lea-Ann S. Kirkham

**Affiliations:** 1 Wesfarmers Centre of Vaccines and Infectious Diseases, Telethon Kids Institute, Perth, Western Australia, Australia; 2 Centre for Respiratory Health, School of Medicine and Pharmacology, University of Western Australia, Perth, Western Australia, Australia; 3 School of Veterinary and Life Sciences, Murdoch University, Perth, Western Australia, Australia; 4 School of Medicine, University of Western Australia, Perth, Western Australia, Australia; 5 Perth Children’s Hospital, Perth, Western Australia, Australia; 6 School of Biomedical Sciences, University of Western Australia, Perth, Western Australia, Australia; Public Health England, UNITED KINGDOM

## Abstract

Otitis media (OM) remains a common paediatric disease, despite advances in vaccinology. Susceptibility to recurrent acute OM (rAOM) has been postulated to involve defective cell-mediated immune responses to common otopathogenic bacteria. We compared the composition of peripheral blood mononuclear cells (PBMC) from 20 children with a history of rAOM (otitis-prone) and 20 healthy non-otitis-prone controls, and assessed innate and cell-mediated immune responses to the major otopathogen nontypeable *Haemophilus* influenzae (NTHi). NTHi was a potent stimulator of inflammatory cytokine secretion from PBMC within 4 hours, with no difference in cytokine levels produced between PBMC from cases or controls. In the absence of antigen stimulation, otitis-prone children had more circulating Natural Killer (NK) cells (p<0.01), particularly NK_dim_ (CD56^lo^) cells (p<0.01), but fewer CD4^+^ T cells (p<0.01) than healthy controls. NTHi challenge significantly increased the proportion of activated (CD107a^+^) NK cells in otitis-prone and non-otitis-prone children (p<0.01), suggesting that NK cells from otitis-prone children are functional and respond to NTHi. CD8^+^ T cells and NK cells from both cases and controls produced IFNγ in response to polyclonal stimulus (Staphylococcal enterotoxin B; SEB), with more IFNγ^+^ CD8^+^ T cells present in cases than controls (p<0.05) but similar proportions of IFNγ^+^ NK cells. Otitis-prone children had more circulating IFNγ-producing NK cells (p<0.05) and more IFNγ-producing CD4^+^ (p<0.01) or CD8^+^ T-cells (p<0.05) than healthy controls. In response to SEB, more CD107a-expressing CD8^+^ T cells were present in cases than controls (p<0.01). Despite differences in PBMC composition, PBMC from otitis-prone children mounted innate and T cell-mediated responses to NTHi challenge that were comparable to healthy children. These data provide evidence that otitis-prone children do not have impaired functional cell mediated immunity.

## Introduction

Otitis media (middle ear infection, OM) is a common childhood disease that is responsible for the greatest number of General Practitioner visits, antibiotic prescriptions, and surgical procedures for children in industrialised countries [1]. Three out of four children will have one episode of OM by the age of 3 years and over 1/3 will have recurring OM, placing a significant burden on healthcare systems [1].

Globally, nontypeable *Haemophilus influenzae* (NTHi) is the most frequently detected bacteria in middle ear of patients with recurrent or chronic OM, and the second most predominant pathogen associated with acute OM after the pneumococcus (*Streptococcus pneumoniae*) [2]. There are conflicting data on whether otitis-prone children have impaired humoral immune responses to OM pathogens and this likely reflects both epidemiological differences and sampling times in different cohorts [3–6]. In our cross-sectional cohort of Australian children under 3 years of age, with and without a history of rAOM, we did not find evidence of reduced circulating antibody to NTHi and pneumococcal antigens [6].

Susceptibility to OM has also been attributed to defective cell-mediated immunity, including reduced NTHi-specific memory CD4^+^ T cells during an active OM episode in children with reduced antibody responses to NTHi antigens [3]. Studies in children with chronic suppurative lung disease (CSLD) and adults with bronchiectasis have revealed impaired CD8^+^ T cell, Natural Killer (NK) cell and interferon-γ (IFNγ) production in response to NTHi challenge [3, 7–11]. As CD8^+^ T cells and NK cells provide a major defence against infection, and there are parallels between CSLD/bronchiectasis and OM in terms of chronic and persistent infection with NTHi, we proposed that otitis-prone children would also have an impaired innate immune responses to NTHi, and that this may contribute to OM susceptibility. Therefore, the aim of this study was to compare cell-mediated and innate immune responses to the major otopathogen, NTHi, in peripheral blood mononuclear cells from otitis-prone and non-otitis-prone children.

## Materials and methods

### Study population

Cryopreserved PBMC samples collected from a cross-sectional study of otitis-prone and healthy children (The GROMIT cohort) were used for this investigation [12]. These cells were cryopreserved at 10^7^ cells/mL using established techniques [13]. Cases (otitis-prone) were defined in terms of clinical phenotype and consist of children aged 3–36 months, who have had 3 or more episodes of acute OM and were undergoing ventilation tube insertion for rAOM. Children who had ventilation tube insertion for OM with effusion only, without previous acute OM episodes, were excluded. Control PBMC samples were collected from healthy, age-matched, clinically immunocompetent individuals, without previous middle ear disease or recurrent infections from a) children admitted to the same hospital for minor surgical procedures and b) children participating as controls in vaccine trials. None of study participants had signs of acute infection at the time of sample collection. Exclusion criteria for both cases and controls were diagnosed immunodeficiency, cystic fibrosis, immotile cilia syndrome, craniofacial abnormalities and genetic syndromes. Data on ear disease and host- and environmental risk factors were obtained from a parental questionnaire and medical records. Study approval was obtained from Princess Margaret Hospital Human Research Ethics Committee (1295_EP). Parents provided informed written consent for their child to participate in this study. Measurements of NTHi carriage [12], respiratory viruses [14] and NTHi-specific serum IgG titres [6] have been previously described for this cohort.

### PBMC stimulation with live NTHi

Two strains of live NTHi (NTHi 86-028NP [15] and NTHi-289 [16]) were used to challenge PBMC from 20 otitis-prone children and 20 age-matched healthy non-otitis-prone children as described previously [17]. Unless otherwise stated, all cell culture reagents were from Gibco, Thermofisher, Australia. Briefly, 200μL PBMC at 1x10^6^ cells/mL in RPMI media (plus 10% Fetal Calf Serum) were incubated at 37°C, 5%CO_2_ with bacteria for 4 hours at a multiplicity of infection of 10:1 (bacteria:cell). The super-antigen Staphylococcal enterotoxin B (SEB, from *S*. *aureus*; Sigma-Aldrich, Australia) was used at 1μg/mL as a positive control and culture media as a negative control (unstimulated) for PMBC stimulation. Following the 4h stimulation, supernatants were collected, filter-sterilised and stored at -80°C for measurement of cytokine production. PBMC were then incubated with 100 μg/mL gentamicin for 1h and then washed in media to prevent bacterial overgrowth in the media. These PBMC were incubated for a further 20h before supernatants were collected, filtered and stored at -80°C. A 48h time-point was also assessed in the optimisation of these experiments but deemed unfeasible due to a >20% increase in cell death (72.8% viability at 48h versus 94.7% viability at 24h), lower frequency of IFNγ+ and IL-13+ cells, and an increase baseline in CD107a expression in unstimulated cells versus a decrease in CD107a expression in SEB stimulated cells.

### Measurement of cytokine levels in supernatants of PBMC challenged with NTHi

IL-5, IL-6, IL-8, IL-10, IL-13, IFNγ, TNFα levels (pg/mL) were measured in cell culture supernatants using a previously described multiplex cytokine bead assay [18] on the Bio-Plex® 200 system (Bio-Rad Laboratories, California, USA). For statistical analyses, samples in which cytokine levels were below the limit of detection were assigned a value that was half of the lowest detectable amount IL-6, and IL-10 = 1.22 pg/mL; IL-8 = 2.54; TNFα = 3.30pg/mL; IFNγ = 2.49pg/mL.

### Multi-parameter flow cytometry

1mL PBMC at 1x10^6^ cells/mL in RPMI media (+10% FCS) were incubated in 5mL round bottom polypropylene tubes with NTHi at a multiplicity of infection of 10:1 (bacteria:cell) for 4h at 37°C, 5% CO_2_. SEB was used at 1μg/mL as a positive control and culture media as a negative control (unstimulated) for PBMC stimulation. Following the 4h stimulation, the PBMC were washed with culture media and 10μg/mL gentamicin was added for 1h to kill extracellular bacteria. PBMC were washed with culture media and replaced with fresh media containing 10 μg/mL brefeldin-A, 6μg/mL monensin and CD107a-PE-Cy7 flow antibody (BD Biosciences, San Jose, CA, USA) and incubated for a further 20h at 37°C in 5% CO_2._ Cells were then washed and stained with fixable viability stain (FVS 620; BD Biosciences) for 10 min at 37°C in 5% CO_2_. Cells were washed twice in 1%(w/v) bovine serum albumin (BSA)/phosphate buffered saline (PBS) and surface stained with CD3-APC-H7, CD4-BB515, CD8-APC and CD56-PE (all BD) for 15 min. Cells were then fixed and permeabilised using the BD Cytofix/Cytoperm kit (BD) according to the manufacturer’s instructions and stained for intracellular IFNγ-BV510 and IL-13-BV421 (BD) for a further 30 min. 200μL of stained cells were then transferred directly into Trucount™ tubes (BD) and 100,000–200,000 events were acquired on a BD FACS Canto II flow cytometer (BD).

Data were analyzed using FlowJo Software version 10 (FlowJo, LLC, OR, USA). Cell doublets and dead cells (FVS positive) were excluded first. Lymphocytes were determined using size and granularity parameters (forward scatter and side scatter areas). Lymphocyte populations were then discriminated based on the surface expression of CD3, CD4, CD8 and CD56. NK cells were further discriminated by population gating on the brightness of the CD56 marker into CD56^lo^ (10^3^–10^4^) and CD56^hi^ (10^4^−10^5^) subsets. Beads from Trucount™ tubes fell into the FVS+ region and were gated on in a separate sub-analysis to enumerate cell populations. Absolute numbers of cells (A) were calculated according to the manufacturer’s protocol by dividing the number of positive cell events in region of interest (X) by the number of Trucount™ bead events (Y) and then multiplying by the Trucount™ bead concentration (N/V, where N = number of beads per sample (pre-determined by bead lot number) and V = sample volume) (A = X/Y × N/V). Absolute cell frequencies were calculated as a percentage of total lymphocyte number (same donor, same treatment) to normalise data. Relative cell frequencies were expressed as a proportion of the parent population.

### Statistics

Host and environmental risk factors were compared between otitis-prone and healthy children using Student’s *t* tests for continuous variables (age and serum IgG titres) and Pearson Chi-square analyses (p-value asymptotic significant 2-sided) for categorical variables (gender, day-care attendance, presence of respiratory virus and NTHi carriage). Mann–Whitney U-tests were performed on non-parametric data sets. Non-parametric one way analysis of variance (ANOVA) (Kruskal-wallis) with post-hoc Dunn’s multiple comparison testing were used to compare multiple data sets. Differences between unstimulated and stimulated samples were analyzed using Wilcoxon signed rank test for paired samples, where p<0.05 was considered significant. Fisher Exact testing was used for categorical analyses of cytokine responses. A p value ≤0.05 was considered statistically significant. The IBM SPSS Statistics 22 for Windows software package (IBM, New York, USA) was used for all statistical analyses and data were plotted using GraphPad Prism 6 (GraphPad Software Inc, California, USA).

## Results

### Study population

Host and environmental risk factors for children in this study are detailed in Table 1. All children in this study were under 3 years of age. Sixty percent of the otitis-prone children (cases) had experienced ≥5 documented episodes of AOM and 30% had experienced ≥8 episodes. Similar proportions of cases and controls had at least one respiratory virus detected in their nasopharynx (88% versus 63%, p = 0.08), whereas most otitis-prone children but no controls were colonised with NTHi at the time of sample collection (85% versus 0%, p <0.0001).

**Table 1:** Demographics and risk factors for otitis prone and healthy children in this study. NTHi, nontypeable *Haemophilus influenzae*; PD, protein D. p<0.05 was considered statistically significant. ^a^The total number of AOM episodes was not recorded for 1 otitis-prone child but they fitted the inclusion criteria of at least 3 doctor-diagnosed episodes of AOM. ^b^Day-care attendance was not recorded for 1 child. ^c^Viral PCR was not conducted on nasopharyngeal (NP) swabs from 3 cases and 1 control. ^d^NP swab was not cultured for 1 control. ^e^No serum IgG data for 2 cases and 1 control.

	Otitis-prone	Healthy	p value
	N = 20	N = 20	
Mean age in months (range)	15.4 (8.5–22.0)	11.4 (3.6–33.4)	0.05
% male	60% (12/20)	80% (16/20)	0.18
# AOM episodes^a^			
3–4	35% (7)	0	-
5–7	30% (6)	0	-
8–9	25% (5)	0	-
10+	5% (1)	0	-
At day-care ≥4h/week	63% (12/19^b^)	10% (2/20)	<0.0001
Virus detected in NP	88% (15/17^c^)	63% (12/19^c^)	0.08
NTHi carriage	85% (17/20)	0% (0/19^d^)	<0.0001
Mean NTHi-specific serum IgG titre (AU/ml +/- SEM)^e^			
P4	269 (+/- 46)	128 (+/- 62)	0.84
P6	1365 (+/- 258)	764 (+/- 283)	0.96
PD	154 (+/- 43)	35 (+/- 8)	0.01

### NTHi is a potent stimulator of innate inflammatory mediators regardless of susceptibility to OM

**Fig 1:**
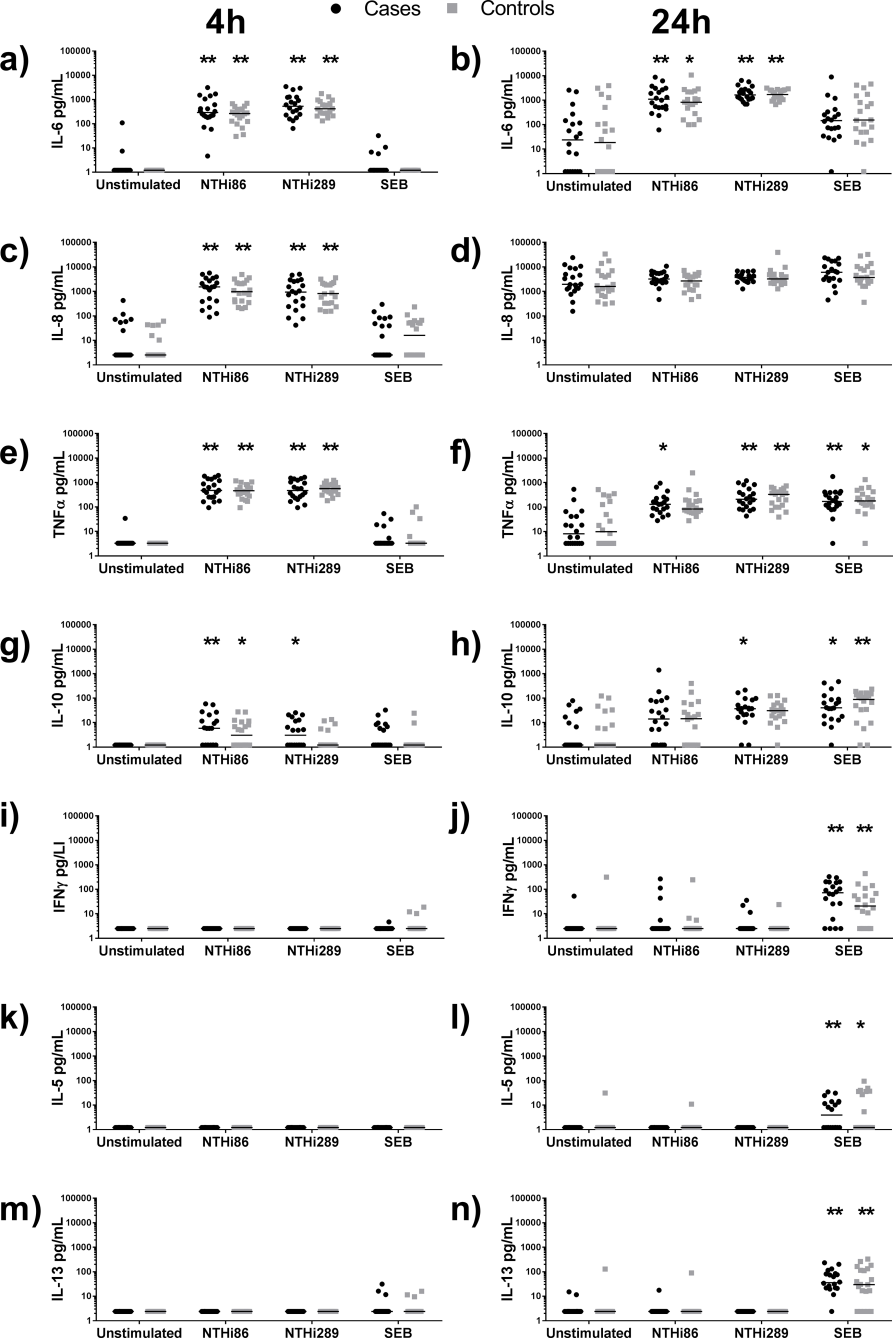
NTHi is a potent stimulator of innate inflammatory mediators regardless of susceptibility to OM. PBMC from otitis-prone children (cases; black dots) or non-otitis-prone children (controls; grey squares) were challenged with NTHi 86-028NP (NTHi-86), NTHi 289 or SEB and cytokine secretion was measured at 4h (left panel) or 24h (right panel) post-stimulation. Each dot represents a child and the horizontal lines represent the median level of cytokine production for each group. ** = p<0.001, * = p<0.05 when each stimulation was compared with unstimulated PBMCs from the same donors.

No differences were observed between cytokine responses from challenged PBMC from otitis-prone children versus non-otitis-prone children (Fig 1). Both strains of NTHi induced early and significant production of pro-inflammatory cytokines IL-6, IL-8 and TNFα from PBMC from cases and controls within 4h of challenge, compared with SEB and untreated cells (Fig 1A, 1C and 1E; p<0.0001). In contrast, SEB, which is a classic polyclonal activator of inflammatory responses, did not induce production of pro-inflammatory cytokines from PBMC of most children by 4h (Fig 1A, 1C and 1E). At 24h post-challenge, IL-6 production from NTHi-challenged PBMC increased from the levels measured at 4h for both cases and controls (Fig 1B), whereas the levels of IL-8 remained similar between the time-points (Fig 1D). Indeed, IL-8 levels were similar from all treatments by 24h, including the unstimulated cells. TNFα levels decreased at 24h post-NTHi challenge (Fig 1F). IL-6, IL-8 and TNFα increased over 24h for both SEB and unstimulated PBMC, however only TNFα was significantly induced by SEB stimulation (p<0.01) and this was the same between cases and controls (Fig 1B, 1D and 1F).

Production of the immunomodulatory cytokines associated with acquired immunity were also assessed (Fig 1G–1N). As with the pro-inflammatory responses, no significant differences in the median levels of immunomodulatory cytokines produced between PBMC from cases and controls were observed. IL-10 release from PBMC was significantly induced by NTHi within 4h (Fig 1G), with levels increasing by 24h (Fig 1H). In contrast to the pro-inflammatory responses, NTHi-induced IL-10 responses were not universal, with some cases and controls remaining non-responders (levels below limit of detection) at 24h post-challenge. Using categorical analyses, no differences were observed in the proportion of IL-10 responders between cases and controls (p = 0.06). IFNγ was produced by PBMC from some cases and controls at 24h after NTHi challenge (Fig 1J), but not within 4h (Fig 1I). SEB challenge of PBMC induced a stronger IFNγ response than NTHi at 24h (Fig 1J). SEB also induced significant production on IL-5 and IL-13 after 24h, but not 4h (Fig 1K–1N). IL-5 and IL-13 PBMC responses were not observed at either time-point following NTHi challenge.

Categorisation of the whole cohort by age into ≤12 months (n = 20) and >12 months (n = 20), to test whether younger children were more likely to be non-responders, revealed that there was no difference in 24h extracellular cytokine levels for IL-5 and IL-13 in response to NTHi or SEB challenge. However, 24h IFNγ levels in SEB-challenged PBMC were significantly higher in children >12 months compared with those ≤12 months of age (median 73 pg/mL in >12 months versus median 21 pg/mL in ≤12 months; p = 0.033). This significant increase in IFNγ in the older children was further analysed by case/control status. We found that it was the younger controls (n = 14) that had lower PBMC IFNγ responses to SEB compared with the older controls (n = 6), p = 0.032 (S1 Table). Whereas the PBMC IFNγ responses to SEB in otitis-prone children was similar regardless of age, p = 0.797. The otitis-prone children ≤12 months of age (n = 6) had a higher IFNγ response to SEB than the age-matched controls (n = 14), 78pg/mL versus 15pg/mL, p = 0.05, demonstrating that PBMC from the otitis-prone children under ≤12 months of age in our study were capable of an IFNγ response. There were no differences observed in PBMC IFNγ responses to NTHi challenge (for both NTHi strains) when young children (≤12 months of age) were compared to children >12 months of age (S1 Table).

### Otitis-prone children did not have a systemic lymphocyte deficiency but had more circulating NK cells and fewer CD4^+^ T cells than non-otitis-prone children

**Fig 2:**
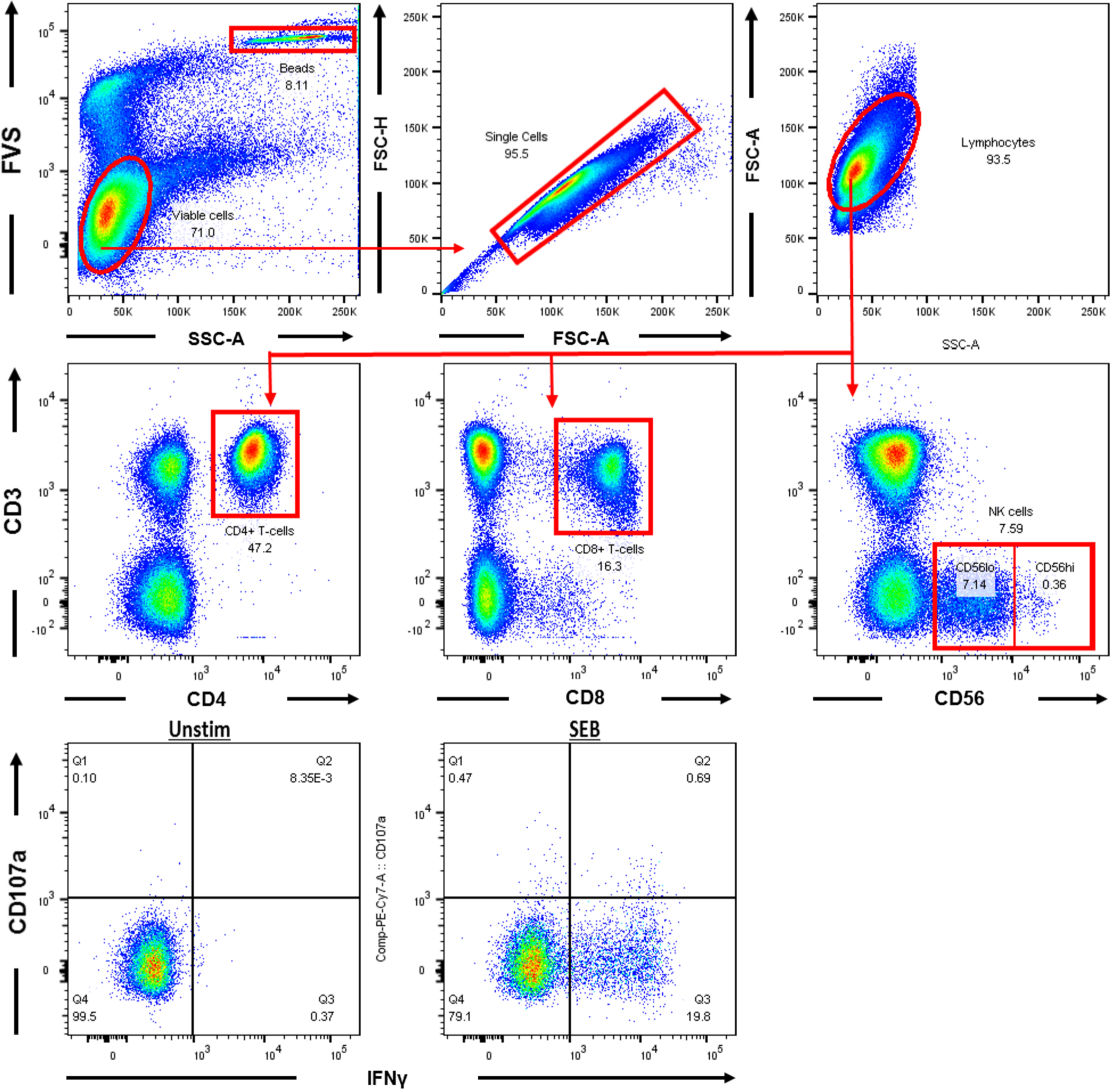
Gating strategy to identify CD4^+^ T cells, CD8^+^ T cells and NK cells. a) Viable cells were gated based on negative expression of the fixable viability stain (FVS). Trucount™ beads (FVS+) were gated on separately (rectangular box in upper right quadrant) to allow enumeration of cell populations. b) Doublets were then excluded and c) lymphocytes were gated based on forward (FSC) and side scatter (SSC) profiles. Within the lymphocyte population, d) CD4^+^ T cells, e) CD8^+^ T cells and f) NK cells were discriminated on the basis of surface expression of CD3 and either CD4, CD8 and CD56 respectively. NK cells were further discriminated by population gating on the brightness of the CD56 marker into CD56^lo^ (10^3^–10^4^) and CD56^hi^ (10^4^−10^5^) subsets (boxes in lower right quadrant). g) A representative plot of expression of CD107a versus IFNγ from unstimulated NK cells versus h) SEB-stimulated NK cells.

The proportion of CD4^+^ or CD8^+^ T cells and NK cells were quantified and their functional responses assessed following treatment with either media alone (unstimulated), two NTHi strains or SEB. The gating strategy used to identity the cell types is shown in Fig 2.

**Fig 3:**
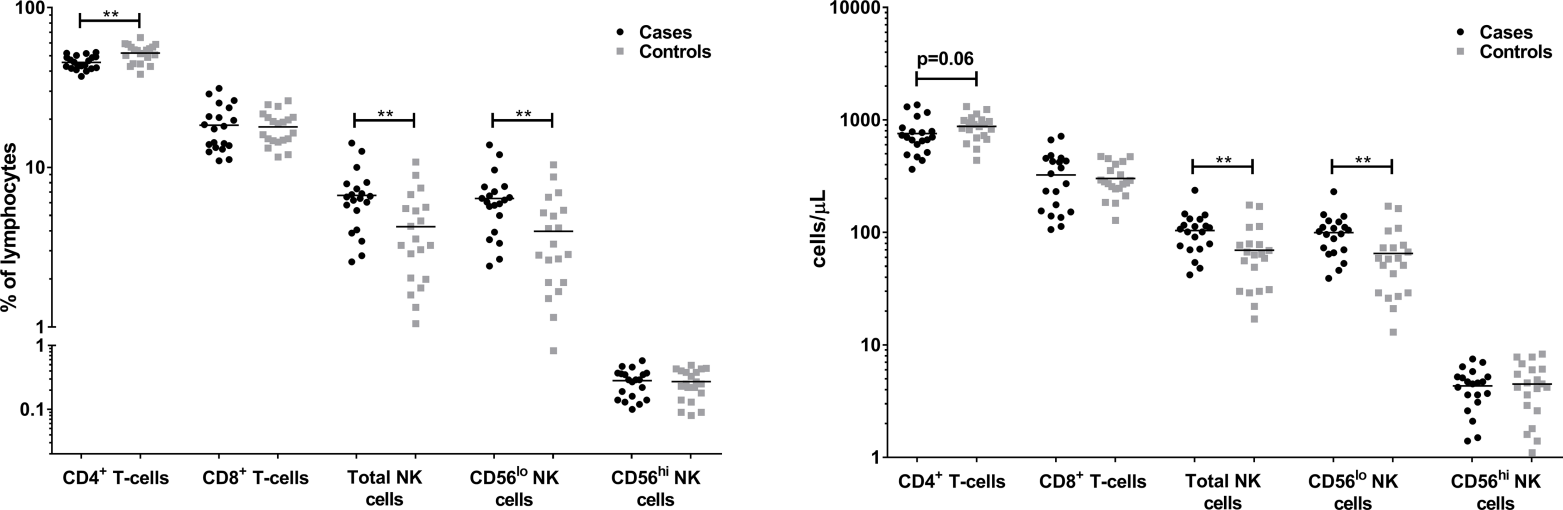
Otitis-prone children have fewer CD4^+^ T cells but more NK cells, particularly CD56^lo^ NK cells, than non-otitis-prone controls. Frequencies of CD4^+^ T cell, CD8^+^ T cell and NK cell (CD56 ^lo^, CD56^hi^) populations are expressed as: a) proportion of the total lymphocytes or b) cells/μL. Horizontal lines represent the median. **p<0.01 when comparing the proportion of cell subsets between cases and controls.

Under steady-state conditions (unstimulated PBMC), otitis-prone children had a lower frequency and number of circulating CD4^+^ T cells (p<0.01) but higher frequency and number of NK cells (p<0.01), while there were no differences in the proportion or number of CD8^+^ T cells between cases and controls (Fig 3). The increased NK cell population is likely due to the increased number of CD56^lo^ NK cells in cases compared to controls (p<0.01), as CD56^hi^ NK cells numbers were similar between groups (Fig 3).

### Otitis-prone children have functional lymphocytes that can be activated in response to stimuli

**Fig 4:**
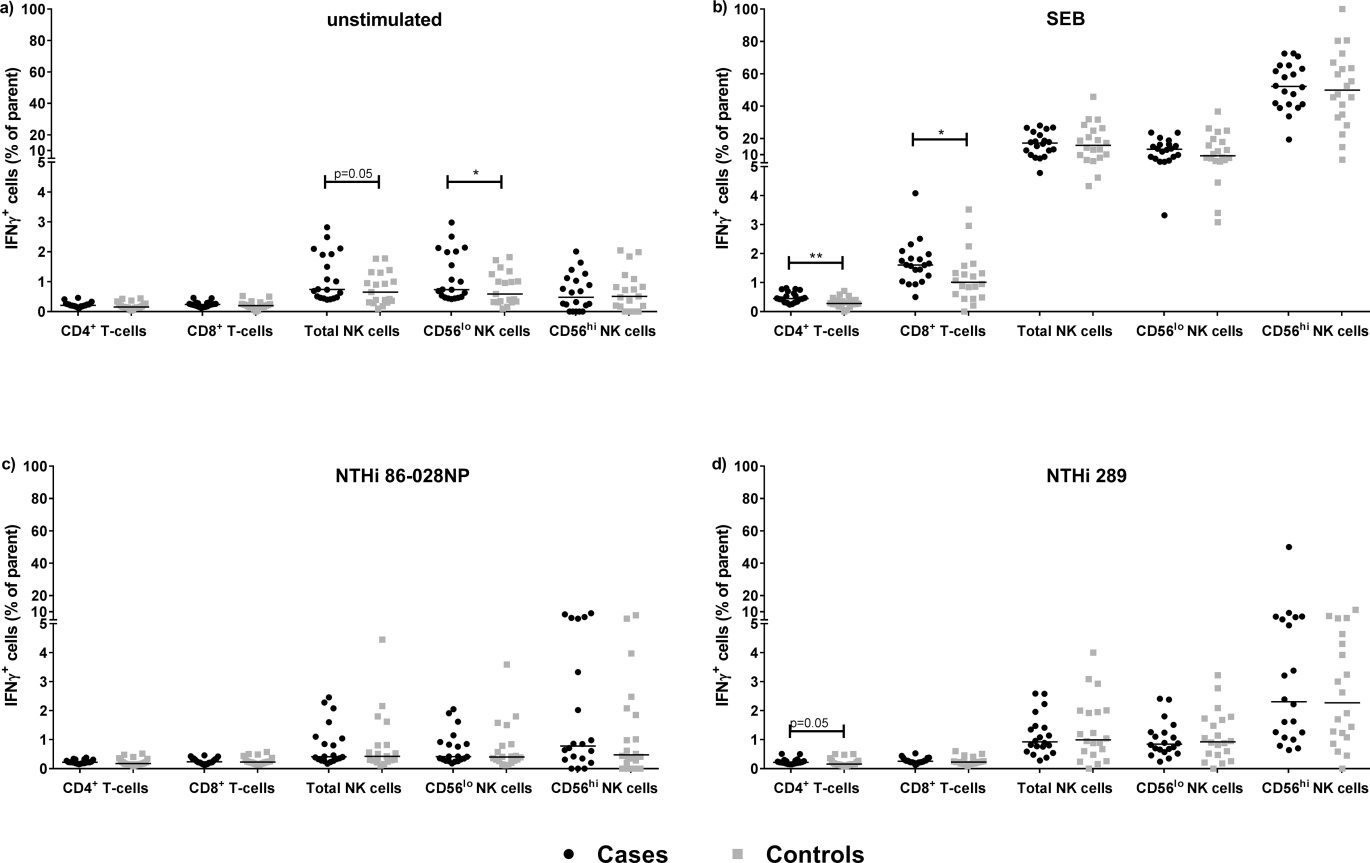
IFNγ^+^ cells were present in PBMC from otitis-prone and non-otitis-prone children following SEB (and to a lesser extent) NTHi challenge. Frequencies of IFNγ producing cells were expressed as a percentage of the parent (CD4^+^ T cell, CD8^+^ T cell or NK cell [CD56^lo^ or CD56^hi^] populations) in: a) unstimulated conditions, b) SEB-stimulated cultures, c) NTHi86-028NP, or d) NTHi289 co-culture. Horizontal lines represent the median. *p<0.05 and **p<0.01 when comparing the proportion of IFNγ^+^ cells between cases and controls.

Intracellular IFNγ and IL-13 production, as well as CD107a expression, was measured in stimulated PBMC as markers of immune cell function. Stimulation with NTHi did not alter the proportion of IFNγ+ cells for any of the lymphocyte populations examined (Fig 4A–4D). Cases had more SEB-activated IFNγ^+^ CD4^+^ T cells and IFNγ^+^ CD8^+^ T cells (Fig 4B) than controls. IFNγ responses in the NK cell subsets did not differ between cases and controls (Fig 4A–4D). IL-13^+^ lymphocytes were not apparent following NTHi challenge (median range of 0.02%– 0.04% IL-13^+^ CD4^+^ T cells), and IL-13^+^ CD4^+^ T cells were detected at very low frequency after stimulation with SEB (median 0.11% IL-13^+^ CD4^+^ T cells following SEB challenge in both cases and controls).

**Fig 5:**
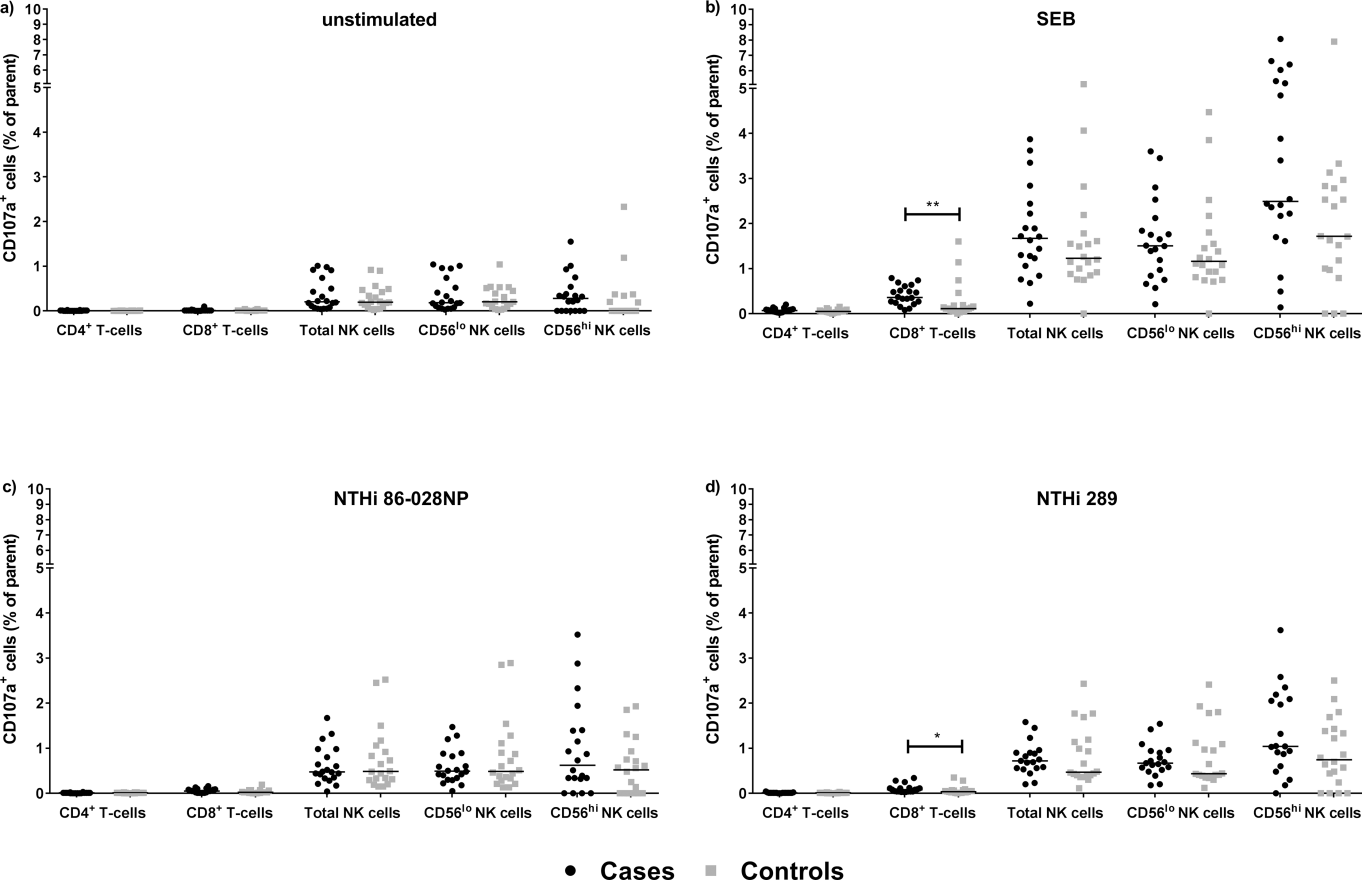
Otitis-prone children have responsive CD8^+^ T cells and NK cells that can express CD107a in response to SEB and NTHi challenge. Frequencies of CD107a-expressing cells were expressed as a percentage of parent (CD4^+^ T cell, CD8^+^ T cell or NK cell [CD56^lo^ or CD56^hi^] populations) in: a) unstimulated conditions, b) SEB-stimulated cultures c) NTHi86-028NP, or d) NTHi289 co-culture. Horizontal lines represent the median. *p<0.05, **p<0.01 when comparing the proportion of CD107a^+^ cells between cases and controls.

CD107a expression is considered a marker of CD8^+^ T cell and NK cell function, allowing identification of activated cells, even in the absence of cytokine secretion. CD8^+^ T cells and NK cells from both cases and controls were found to express CD107a in response to SEB (Fig 5B) and challenge with both NTHi strains (Fig 5C and 5D). Compared to healthy controls, otitis-prone children had small but significantly increased frequencies of CD107a^+^ CD8^+^ T cells in response to SEB (p<0.01, Fig 5B), NTHi86-028NP (p = 0.06, Fig 5C), and NTHi289 (p<0.05, Fig 5D). Evidence of activated NK cells in response to SEB and NTHi was also evident compared to unstimulated cells but there was no significant difference between cases and controls in the proportion of CD107a^+^ NK cells (Fig 5). SEB appeared to be a more potent activator of CD107a expression by NK cells than NTHi at 24h post challenge. Increased CD107a expression following SEB treatment was observed in both CD56^lo^ and CD56^hi^ NK cells (Fig 5B) compared with unstimulated cells (Fig 5A), p<0.01 and p<0.05 respectively.

When we stratified our cohort by day-care attendance rather than OM status, we found that the proportion of circulating NK cells, particularly activated CD107a+ cells, was also significantly higher in unstimulated PBMC from the 14 children attending day-care for ≥4h per week compared to the 25 children attending <4h of day-care per week (9.22% versus 5.90% NK cells in total lymphocytes; p<0.05, and 0.14% versus 0.02% CD107a+ NK cells in total lymphocytes; p<0.001). Day-care attendance and development of recurrent otitis media are inextricably linked, however, assessment of NK cell populations in the 25 children in our study who did not attend day-care (7 otitis-prone versus 18 non-otitis prone) revealed that otitis-prone non-day-care attendees still had a higher proportion of NK cells compared to their non-otitis-prone counterparts (9.2% versus 5.8% NK cells as % total lymphocytes), p = 0.107. Whilst this difference was not statistically significant it suggests that the NK cell effect we observed in our otitis-prone cohort is independent of day-care attendance.

## Discussion

This cross-sectional study is the first to indicate that otitis-prone children do not have an inherent functional deficiency within their circulating immune cells. We have demonstrated that NTHi is a potent driver of innate immunity through production of pro-inflammatory mediators by immune cells, and that this response to NTHi is similar in otitis-prone and non-otitis-prone children.

Host immunity to NTHi, particularly in the context of recurrent ear disease, is not well understood but like other pathogens, requires coordinated responses of both the innate and adaptive immune system. Studies using murine lung infection models have demonstrated that TLR4-dependant production of pro-inflammatory cytokines, including IL-6 and TNFα, is critical for the control and clearance of NTHi [19, 20]. PBMC from children with CSLD have been found to produce less IL-6 and IFNγ in response to live NTHi challenge than healthy controls [21], but this was not apparent in the otitis-prone children in our study. The cell types associated with IFNγ deficiency in children with CSLD have not been identified, but King *et al*. showed reduced production of IFNγ from both CD8^+^ T cells and NK cells in response to challenge with heat-killed NTHi [8]. On stimulation with NTHi, we observed low levels of IFNγ secretion and few IFNγ producing lymphocytes, however, when stimulating with the potent mitogen, SEB, IFNγ was produced at high levels by both cases and controls. As cytokine levels by stimulated PBMC were comparable between otitis-prone and healthy children, we suggest that otitis-prone children, unlike CSLD and bronchiectasis patients, can mount appropriate innate immune responses to NTHi. Therefore, the failure of otitis-prone children to effectively clear otopathogens such as NTHi may not be attributed to systemic deficiencies in cytokine production.

It has been postulated that OM susceptibility is due to impaired T cell-mediated immunity [10, 11, 22], with one study demonstrating reduced IFNγ^+^ memory CD4^+^ T cells in PBMC from otitis-prone children following challenge with recombinant NTHi antigens (but not SEB). Importantly, antigen-specific IgG responses to NTHi antigens were also reduced in this cohort and the authors speculate that this resulted from poor CD4^+^ T cell help [10]. Contrary to this, in our cohort we demonstrated that although there are fewer circulating CD4^+^ T cells in otitis-prone children, the frequency of IFNγ producing CD4^+^ T cells did not differ between otitis-prone and non-otitis-prone children in response to either live NTHi or SEB challenge. IgG responses to NTHi antigens were also not deficient in the otitis-prone children in our study [6]. These conflicting findings can be attributed to a number of factors. Firstly, blood samples from the otitis-prone children in our cohort were collected when participants were convalescent [12], whereas in the study where deficient T-cell mediated immunity was observed the otitis-prone children had blood collected during a confirmed episode of active infection [10]. It is possible that in children currently experiencing an episode of OM, the reduced IFNγ^+^ CD4^+^ T cell response to NTHi challenge is a reflection of lymphocyte exhaustion (or deployment of these antigen-reactive cells to the ear) rather than an inherent cell-mediated immunodeficiency [23]. Secondly, we used live bacteria rather than purified protein antigens to challenge the PBMC. Live bacteria provide a more potent and biologically relevant immunostimulation of PBMC and engage both innate and adaptive immune resposnes [8, 17, 21]. We acknowledge that the 24h time-point in this study may not represent the peak response for all cytokines in response to NTHi challenge. However, cell viability was compromised at a 48h post-challenge with live NTHi (a limitation of using live bacteria for PBMC challenge studies). The 24h time-point chosen represents a necessary compromise for cytokine production and cell viability.

Our observation that otitis-prone children have fewer CD4^+^ T cells but more circulating NK cells, predominantly the activated CD56^lo^ NK cells, in comparison with healthy controls has not, to our knowledge, been reported before. We speculate that exposure to respiratory pathogens (e.g. from chronic upper respiratory tract infection or persistent exposure in a day-care setting) is responsible for the elevation of activated circulating NK cells in the otitis-prone (or day-care attending) children. Indeed, a study of children with frequent upper respiratory tract infections found that they had elevated levels of circulating NK cells in comparison with healthy age-matched controls [24]. While the consequences of elevated levels of circulating NK cells are unknown, there are arguments to support both damaging and beneficial effects [25–29]. It is also plausible that the increased NK cell number reflects persistence of NK cells and possibly NK cell memory, for which there is emerging evidence from animal models that antigen-specific NK cell memory can influence long term immunological outcomes [26, 27, 29]. The role of effector and memory NK cell responses to NTHi in recurrent and chronic ear infection remains to be determined.

CD56^lo^ NK cells are the most efficient NK cells at directing cellular cytotoxicity [30, 31]. Challenge of PBMC with NTHi resulted in strong activation of cytotoxic CD107a^+^ CD56^lo^ NK cells, despite the lack of IFNy production. The relative proportions of CD107a^+^ NK cells was similar between otitis-prone and non-otitis-prone children, suggesting that even with greater numbers of NK cells, the functional activity of NK cells is not different in otitis-prone children. In contrast to our observations, NTHi challenged PBMC from adults with bronchiectasis produced less IFNγ but equal proportions of CD107a^+^ NK cells (and CD107a^+^ CD8^+^ T cells) in comparison with PBMC from healthy adult controls [8].

In summary, our investigation into innate and cell-mediated immune responses to NTHi in otitis-prone versus non-otitis-prone children revealed differences in PBMC composition but not PBMC responses to the otopathogen NTHi. This supports our previous finding of functional humoral immune responses to NTHi in otitis-prone children [6]. However, it is important to note that other cohorts of otitis-prone children have been shown to have impaired humoral and cell-mediated immunity to NTHi antigens [4, 11, 32]. This disparity demonstrates the complexity of OM and suggests that specific population differences exist, highlighting the importance of understanding immune responses to otopathogens in different populations, and throughout the disease course, as this may have downstream effects on development of effective preventions and therapeutics. We conclude that the otitis-prone children in our study did not have impaired cell-mediated immune responses to the major otopathogen NTHi.

## Supporting information

S1 Table Median PBMC IFNγ responses (pg/mL +/- range) assessed by age and otitis media status. (DOCX)
