# Supplementary material for: Evidence of functional cell-mediated immune responses to nontypeable Haemophilus influenzae in otitis-prone children
Source: PLoS One. 2018 Apr 5;13(4):e0193962. doi: 10.1371/journal.pone.0193962 (PMC5886403; doi:10.1371/journal.pone.0193962)
Supplement: S1 Table — (DOCX) [file pone.0193962.s001.docx]

**Supplementary Table 1.**

**Median PBMC IFNγ responses (pg/mL +/- range) assessed by age and otitis media status**

|  | **≤12 m controls** | **>12m controls** | **p** | **≤12 m cases** | **>12m cases** | **p** |
| --- | --- | --- | --- | --- | --- | --- |
|  | N = 14 | N = 6 |  | N = 6 | N = 14 |  |
| **NTHi 86** | 2 (2-246) | 2 (2-7) | 0.840 | 2 (2-5) | 2 (2-268) | 0.662 |
| **NTHi 289** | 2 (2-2) | 2 (2-24) | 0.300 | 2 (2-2) | 2 (2-36) | 0.521 |
| **SEB** | 15 (2-109) | 105 (2-440) | 0.032 | 78 (2-188) | 70 (2-329) | 0.797 |
